# FEATURES OF THE SOCIAL AND PHYSICAL ENVIRONMENT AND ACCELEROMETRY-BASED PHYSICAL ACTIVITY AMONG OLDER ADULTS

**DOI:** 10.1093/geroni/igad104.0543

**Published:** 2023-12-21

**Authors:** Erica Twardzik, Vicki Freedman, Pablo Martinez-Amezcua, Jennifer Schrack

**Affiliations:** Johns Hopkins Bloomberg School of Public Health, Baltimore, Maryland, United States; University of Michigan, Ann Arbor, Michigan, United States; Johns Hopkins Bloomberg School of Public Health, Baltimore, Maryland, United States; Johns Hopkins University, Baltimore, Maryland, United States

## Abstract

Social and physical environments shape physical activity (PA) opportunities among older adults. Whether environmental factors are associated with patterning of PA among older adults is less understood. We examined associations of the social and physical environment with accelerometry-based daily PA metrics among older adults. Cross-sectional data from 2021 National Health and Aging Trends Study respondents with non-missing data were included. Using linear regression models with analytic survey weights and adjusted for potential confounders we investigated the associations between four environmental features: social cohesion, neighborhood disorder, broken step into the home, and continuous sidewalks with PA patterns. Three accelerometry-based PA metrics were examined: total activity counts, PA fragmentation, and number of PA bouts. 633 older adults (45% female) were included in our analytic sample. Respondents with a broken step into their home, compared with those without, had lower total activity counts (♌=-170024.3, SE=81530.6, p<.05). Compared to those living in a neighborhood with no disorder, those living in a neighborhood with any disorder had more PA bouts per day (♌=10.5, SE=3.9, p<.01). All other contextual factors examined were not associated with activity counts, fragmentation, or bouts of PA. Greater physical barriers to access the neighborhood may explain why broken steps were associated with less total PA (volume and intensity). Neighborhood disorder was associated with number of PA bouts per day, which may be due to greater reliance on walking for transportation in disordered neighborhoods. Future work should investigate how the neighborhood environment is structured (e.g., walkability) to elucidate these findings.

